# High-Throughput Screening for Ligands of the HEPN Domain of Sacsin

**DOI:** 10.1371/journal.pone.0137298

**Published:** 2015-09-14

**Authors:** Xinlu Li, Marie Ménade, Guennadi Kozlov, Zheping Hu, Zheng Dai, Peter S. McPherson, Bernard Brais, Kalle Gehring

**Affiliations:** 1 Department of Biochemistry, McGill University, Montreal, Quebec, Canada; 2 Groupe de recherche axé sur la structure des protéines, Montreal, Quebec, Canada; 3 Department of Neurology and Neurosurgery, Montreal Neurological Institute, McGill University, Montreal, Quebec, Canada; University of Saskatchewan, CANADA

## Abstract

Sacsin is a large protein implicated in the neurodevelopmental and neurodegenerative disease autosomal recessive spastic ataxia of Charlevoix-Saguenay (ARSACS), which features the loss of Purkinje neurons in the cerebellum. Although the domain architecture of sacsin suggests that it is a neuronal chaperone assisting in protein quality control, the precise function of sacsin remains elusive. Using fluorescence polarization (FP) assays, we confirmed that the HEPN domain of sacsin binds to nucleotides with low micromolar affinities. FP competition assays with a variety of nucleotides and nucleotide analogs revealed that the binding is primarily mediated by the phosphate groups of nucleotides. A high-throughput screen subsequently identified novel small molecule ligands of HEPN, providing new chemical probes for cell culture studies and drug development. Together, the results are consistent with the HEPN domain contributing to the functional activity of sacsin by binding to nucleotides or other multiply charged anionic compounds in neurons.

## Introduction

Mutations in sacsin, a large 4579 amino acid protein, are causative of the neurodevelopmental and neurodegenerative disease autosomal recessive spastic ataxia of Charlevoix-Saguenay (ARSACS). The disease is primarily characterized by the degeneration of cerebellar Purkinje neurons, the spinal cord and a peripheral neuropathy resulting in symptoms such as spasticity, ataxia and distal muscle wasting [1]. The disease-associated gene (*SACS*) was discovered by Engert *et al*. in 2000, which allowed for molecular identification of the disease in a large number of patients [2]. ARSACS is currently the second most common recessive ataxia in the world, with over 170 different *SACS* mutations identified in patients from more than 20 countries [3].

The domain architecture of sacsin suggests that it is a chaperone contributing to protein quality control. At its N-terminus, Sacsin contains a ubiquitin-like (UbL) domain that was shown to interact with the 26S proteasome and mediate protein degradation [4]. The bulk of the protein, over 80% of the amino acids, consists of three large repeating regions named sacsin internal repeats (SIRPTs). Each SIRPT contains a region homologous to the ATPase domain of Hsp90 chaperone [5]. A purified N-terminal fragment of sacsin including the UbL, SIRPT1, and the linker before SIRPT2 was shown to harbor ATPase and chaperone activities [6, 7]. An additional chaperone domain in sacsin, DnaJ, was able to functionally replace the bacterial Hsp40 chaperone *in vivo* [4]. The C-terminus of sacsin contains a higher eukaryotes and prokaryotes nucleotide-binding domain (HEPN), which is found in a large number of bacterial, yeast and higher eukaryotic proteins, and acts as nucleotide- or RNA-binding domain in diverse contexts [8, 9]. Kanamycin nucleotidyl-transferase, which contains a C-terminal HEPN domain, mediates bacterial resistance to kanamycin by removing a nucleoside mono-phosphate group from the antibiotics [10, 11].

Cell culture studies and a knockout (KO) mouse model implicate sacsin in the maintenance of mitochondrial fission/fusion dynamics. Both ARSACS patient-derived cell lines and sacsin-knockdown (KD) neuronal cultures show enlarged and hyperfused mitochondria with dissipated mitochondrial membrane potential and altered localization [12]. The sacsin-KO mouse, which recapitulates many of the clinical manifestations found in ARSACS patients, exhibits similar neuronal mitochondrial phenotype to those observed in the cell culture studies. Furthermore, immunolabeling and western blots revealed abnormal bundles of non-phosphorylated neurofilament (npNFH) in sacsin-deficient neurons, which could disrupt mitochondrial transport [13].

Several potential interacting partners of sacsin have been identified, providing clues to its functional mechanism. Co-immunoprecipitation in neuronal culture showed that sacsin interacts with dynamin-related protein 1 (Drp1), a small GTPase required for mitochondrial fission [12]. A yeast two-hybrid screen identified four additional interacting partners of sacsin, namely tRNA(His) guanylyltransferase (THG-1), DNA-directed RNA polymerase I subunit Rpa12, α-actinin 4 and PRKCA-binding protein (PICK1) [14]. Of particular interest is α-actinin 4, which is involved in actin filament bundling and could contribute to the mitochondrial phenotype of the sacsin-KO mouse. However, the direct interactions between sacsin and these proteins, as well as their physiological relevance, remain to be validated.

In this study, we sought to understand the function of sacsin by studying its C-terminal HEPN domain. We previously determined the structure of the sacsin HEPN domain and showed that it forms a dimer in solution using size exclusion chromatography-multiangle light scattering and nuclear magnetic resonance (NMR) translational self-diffusion experiments [15]. In the crystal structure, two malonate ions (present at 0.2 M in the crystallization solution) were found in a symmetric, positively charged pocket at the dimerization surface. Isothermal titration calorimetry (ITC) and NMR titration experiments revealed that nucleotides bind with micromolar affinity to the HEPN domain. Assignment of the NMR signals confirmed the symmetry of the dimer and mapped nucleotide binding to the malonate-binding site observed in the crystal structure.

To further characterize the substrate-binding properties of HEPN and potentially identify novel ligands we developed a fluorescence polarization (FP) assay. The assay confirmed that HEPN binds to nucleotides and nucleotide analogs with high affinity. HEPN prefers nucleotides with larger numbers of phosphates, which suggests that the binding is driven by electrostatic interactions. Both Mg^2+^ and EDTA disrupt the binding of nucleotides. The FP assay was subsequently developed into a high-throughput screen, which identified three novel ligands that can be used to investigate sacsin’s cellular function in biochemical or cell culture studies.

## Materials and Methods

### HEPN expression and purification

The HEPN domain (residues 4441–4579) was purified as in Kozlov *et al*. [15]. Briefly, HEPN in pGEX-4T-1 was expressed in the *E*. *coli* cell line BL21 in LB medium and purified as an 84 kDa GST-tagged protein with glutathione Sepharose resin. For FP assays, the fusion protein was further purified with size-exclusion chromatography and migrated as a single band on SDS-PAGE. For NMR experiments, HEPN was ^15^N-labeled by culturing the BL21 cells in M9 minimal medium with ^15^NH_4_Cl and purified as a GST-tagged protein. The GST-tag was removed by overnight incubation with thrombin at 4°C, after which the cleaved protein was further purified by size exclusion chromatography [15]. Final protein concentrations (as monomers) were determined by UV absorbance at 280 nm using extinction coefficients of 61310 M^−1^·cm^−1^ for GST-HEPN and 18450 M^−1^·cm^−1^ for HEPN.

### Fluorescently labeled ATP probes for FP assays

*N*-methylanthraniloyl 8[(4-amino)butyl]-amino-ATP (MABA-ATP) and fluorescein-12-ATP (F12-ATP) were purchased from Jena Bioscience and PerkinElmer, respectively. The excitation and emission wavelengths of MABA-ATP were reported to be 335 nm and 440 nm, and those of F12-ATP were reported to be 496 nm and 517 nm by the commercial suppliers. The optimal excitation and emission wavelengths were determined under FP assay conditions using 0.2 μM MABA-ATP and 3.3 nM F12-ATP in 100 mM NaCl, 20 mM Bis-tris, pH 6.5.

### FP titration assay

A fixed concentration of the probe (0.5 μM MABA-ATP or 5 nM F12-ATP) was put in each well followed by a serial dilution of GST-HEPN starting at 10 μM. The assay was conducted in 20 mM Bis-tris pH 6.50 and 100 mM NaCl and the final volume in each well was 100 μl. The total fluorescence and fluorescence polarization signals of each well were recorded on a SpectraMax M5e Microplate Reader at the optimal excitation and emission wavelengths of the probes. A titration curve was generated by plotting the fluorescence polarization signals as milli-polarization units (mP) against a log scale of GST-HEPN concentration (μM).

### FP competition assay

In FP competition assays, a fixed concentration of the probe and GST-HEPN (5 nM F12-ATP and 120 nM GST-HEPN or 0.5 μM MABA-ATP and 7 μM GST-HEPN) were first put into each well. The probe concentration was chosen to be the minimum concentration required to generate a sigmoidal binding curve. A value between 50% and 80% of the Kd in the titration assay was selected for the GST-HEPN concentration to be used. Next, a serial dilution of the competitor compounds (nucleotides and nucleotide analogs) starting at 10 mM were added. The fluorescence polarization signal in each well was measured on a SpectraMax M5e Microplate Reader.

Affinities of the compounds for GST-HEPN were calculated according to equation 51 in Roehrl *et al*. [16]:

$$R_{3}=\frac{[–L_{ST}F_{SB}^{2}+(K_{D1}+L_{ST}–L_{T}+R_{T})F_{SB}+L_{T}–R_{T}]F_{SB}K_{D1}}{\left(1–F_{SB})[L_{ST}F_{SB}^{2}–(K_{D1}+L_{ST}+R_{T})F_{SB}+R_{T}\right]}$$

where *R*_3_ denotes the compound affinity for GST-HEPN in nM, *L*_*ST*_ the probe/F12-ATP concentration (5 nM), *R*_*T*_ the ligand/GST-HEPN concentration (120 nM), *F*_*SB*_ the fraction of probe/F12-ATP bound to the ligand/GST-HEPN (0.35 at midpoint of the competition curve), *K*_D1_ the dissociation constant (78 nM) between the probe (F12-ATP) and the ligand (GST-HEPN), and *L*_*T*_ the concentration of the competitor compound at midpoint of the titration curve (in nM) [16].

### FP high-throughput screen

The FP high-throughput screen was performed at the High-throughput Screen Facility at Université de Montréal. The screen was conducted in 384-well formats with 5 nM F12-ATP and 120 nM GST-HEPN in 20 mM Bis-tris pH 6.50 and 70 mM NaCl. 15 μl of GST-HEPN was first added to each well and 160 nl of compounds (15 μM) was added with Pin-tool. The total fluorescence of each well was read to detect compounds with auto-fluorescence. 10 μl of F12-ATP was subsequently added and the FP signal was read at excitation and emission wavelengths of 490 nm and 520 nm, respectively. The counter screen was conducted with the same setup as the primary screen with 600 nM of GST-MLLE (the C-terminal domain of cytoplasmic poly(A) binding protein 1, PABPC1) and 10 nM of a PAM2 peptide (PABP-interacting motif 2) labeled with fluorescein isothiocyanate in 10 mM HEPES pH 7.4, 150 mM NaCl, 3mM EDTA and 0.05% (v/v) Tween-20.

### NMR titrations

Between 100 μM and 400 μM ^15^N-HEPN were used for NMR titration experiments. Heteronuclear single quantum correlation (HSQC) spectra were acquired in 20 mM Bis-tris pH 6.50 and 70 mM NaCl at 303 K using Varian 800 MHz or Bruker 600 MHz NMR spectrometers. The data were processed by NMRPipe. NMR spectra were then generated and analyzed by NMRView. The 2D HSQC spectra were overlaid with previously assigned spectrum of HEPN. The combined chemical shift perturbations in the ^1^H and ^15^N dimensions of each peak, calculated as (Δδ*H* ^2^ + (0.2 × Δδ*N*)^2^)^0.5^, were plotted against the residue number. Residues near the GTP-binding site that showed changes greater than 0.05 ppm were labeled and mapped onto the HEPN crystal structure (PDB: 3O10).

## Results

### Fluorescence Polarization Assay Development

**Fig 1:**
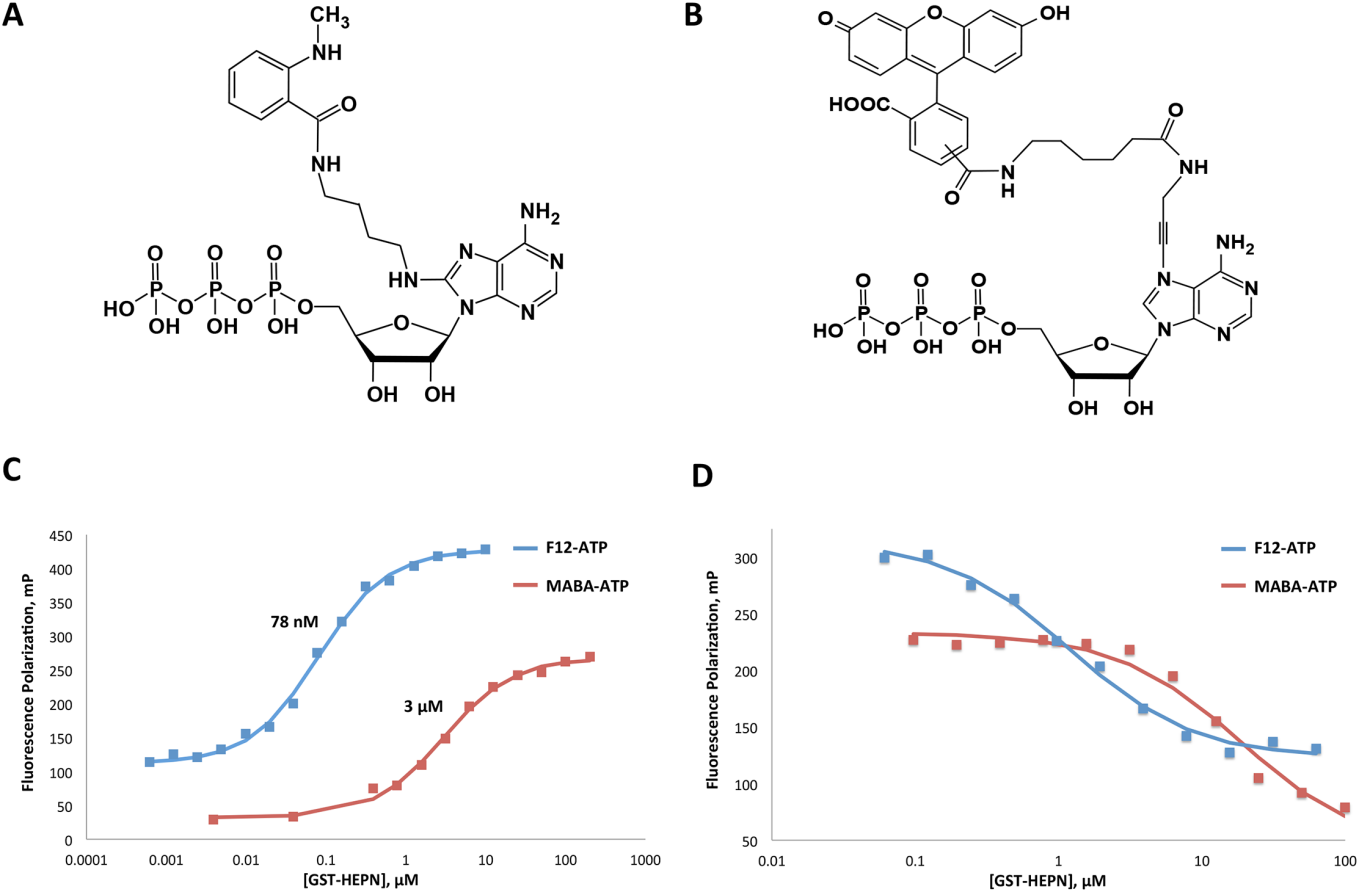
Fluorescence polarization assay development and validation. A. Chemical structure of 8[(4-amino)butyl]-amino-ATP-MNT (MABA-ATP). B. Chemical structure of fluorescein-12-ATP (F12-ATP). C. 0.5 μM MABA-ATP and 5 nM F12-ATP were titrated against various concentrations of GST-HEPN. D. 0.5 μM MABA-ATP and 5 nM F12-ATP and 120 nM / 7 μM GST-HEPN were titrated against various concentrations of GTP to verify that the probes bind to HEPN at its nucleotide-binding site.

Two fluorescently labeled ATP compounds, MABA-ATP and F12-ATP (Fig 1A and 1B), were chosen to develop the fluorescent polarization assay. The optimal excitation and emission wavelengths of the probes were determined to be 338 nm and 436 nm for MABA-ATP, and 490 nm and 526 nm for F12-ATP (S1 Fig). MABA-ATP and F12-ATP were titrated against various concentrations of GST-HEPN (Fig 1C). The binding affinities of the probes were estimated from the titration curves to be approximately 3 μM for MABA-ATP and 78 nM for F12-ATP. Titration of the probes with GST alone confirmed that GST does not bind to either probe (S2 Fig). Competition assays were subsequently performed with fixed concentrations of MABA-ATP/ F12-ATP and GST-HEPN titrating against various concentrations of GTP (Fig 1D). Both probes were effectively displaced from HEPN by GTP, confirming that the probes interact with the HEPN nucleotide-binding site. The affinity of GTP for the sacsin HEPN domain was estimated to be 0.81 μM, which is similar to the value previously determined by isothermal titration calorimetry (ITC) [15].

F12-ATP was selected for subsequent FP assays due to its superior characteristics. F12-ATP has longer excitation/emission wavelengths, higher binding affinity and a greater FP signal change upon binding GST-HEPN than MABA-ATP. In FP assays, the two main sources of interference are compound fluorescence and light scattering due to aggregation: both are more pronounced at shorter wavelengths [17]. The high affinity between F12-ATP and GST-HEPN allows for better sensitivity and resolution in FP competition assays. The competition assays with F12-ATP allowed us to minimize the concentrations of reagents present and potential artifacts due to protein aggregation [18].

### Identifying the F12-ATP-binding site on HEPN

**Fig 2:**
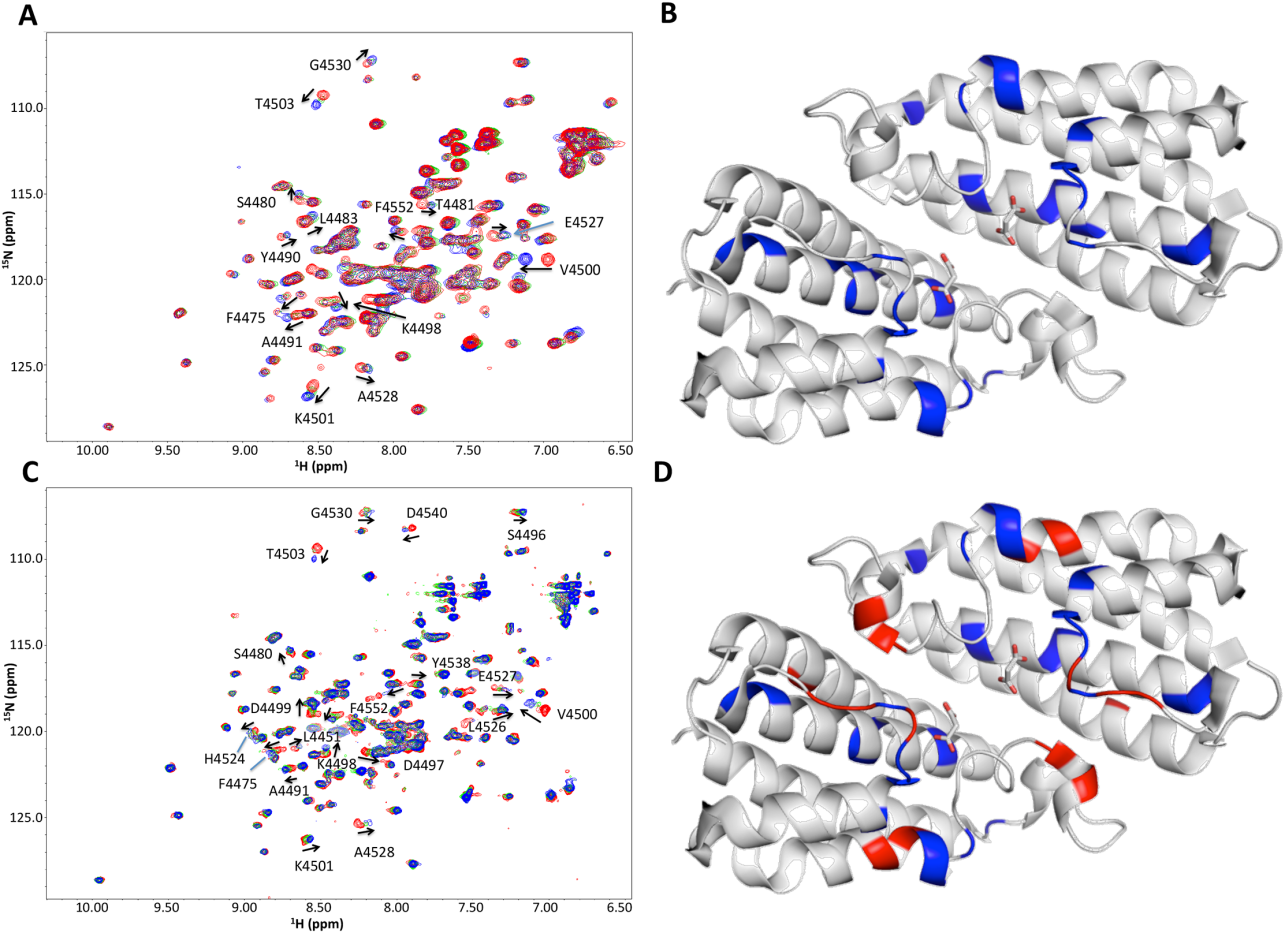
Characterizing the interaction between F12-ATP and GST-HEPN. A. Overlay of ^15^N,^1^H-HSQC NMR spectra of 200 μM ^15^N-HEPN alone (*red*) and following the addition of GTP (50 μM GTP in *green* and 100 μM in *blue*). Residues that showed chemical shift perturbations in the presence of GTP were identified from the assigned HEPN spectrum and labeled. B. Residues involved in GTP-binding are mapped onto the HEPN crystal structure (PDB: 3O10) and labeled in *blue*. Malonate ions present in the crystal structure are presented in stick representation, with their oxygen atoms labeled in *red*. C. Overlay of ^15^N,^1^H-HSQC NMR spectra of 110 μM ^15^N-HEPN alone (*red*) and following the addition of F12-ATP (55 μM in *green* and 110 μM in *blue*). D. Residues involved in F12-ATP-binding are mapped onto the HEPN crystal structure. Residues involved in both F12-ATP and GTP-binding are labeled in *blue* and additional residues that shifted during the F12-ATP titration are labeled in *red*.

We proceeded to investigate the binding between F12-ATP and HEPN using an ^1^H-^15^N HSQC NMR titration experiment. ^15^N-HEPN was first titrated with GTP as a control (Fig 2A). The NMR chemical shift perturbation of each peak was plotted against the residue number (S3A Fig). Mapping the chemical shift perturbations onto the HEPN crystal structure confirmed that GTP binds to HEPN at its dimerization surface (Fig 2B). Since F12-ATP binds to GST-HEPN with about 40-fold higher affinity than ATP and GTP, we hypothesized that the fluorescein group of F12-ATP forms additional contacts with HEPN. ^15^N-HEPN was titrated against F12-ATP (Fig 2C). F12-ATP interacts with all HEPN residues involved in GTP-binding. The locations of the shifted residues on the HEPN crystal structure revealed that the F12-ATP interacts more extensively with the loops at the GTP-binding pocket and induces chemical shift perturbations in residues on the outer surface of the pocket (S3B Fig and Fig 2D). The fluorescein group of F12-ATP likely enhances the HEPN-binding affinity of ATP by orienting the latter in a more favorable geometry for binding and forming additional contacts with residues adjacent to the GTP-binding pocket.

### Mg^2+^ and EDTA both disrupt binding between F12-ATP and HEPN

**Fig 3:**
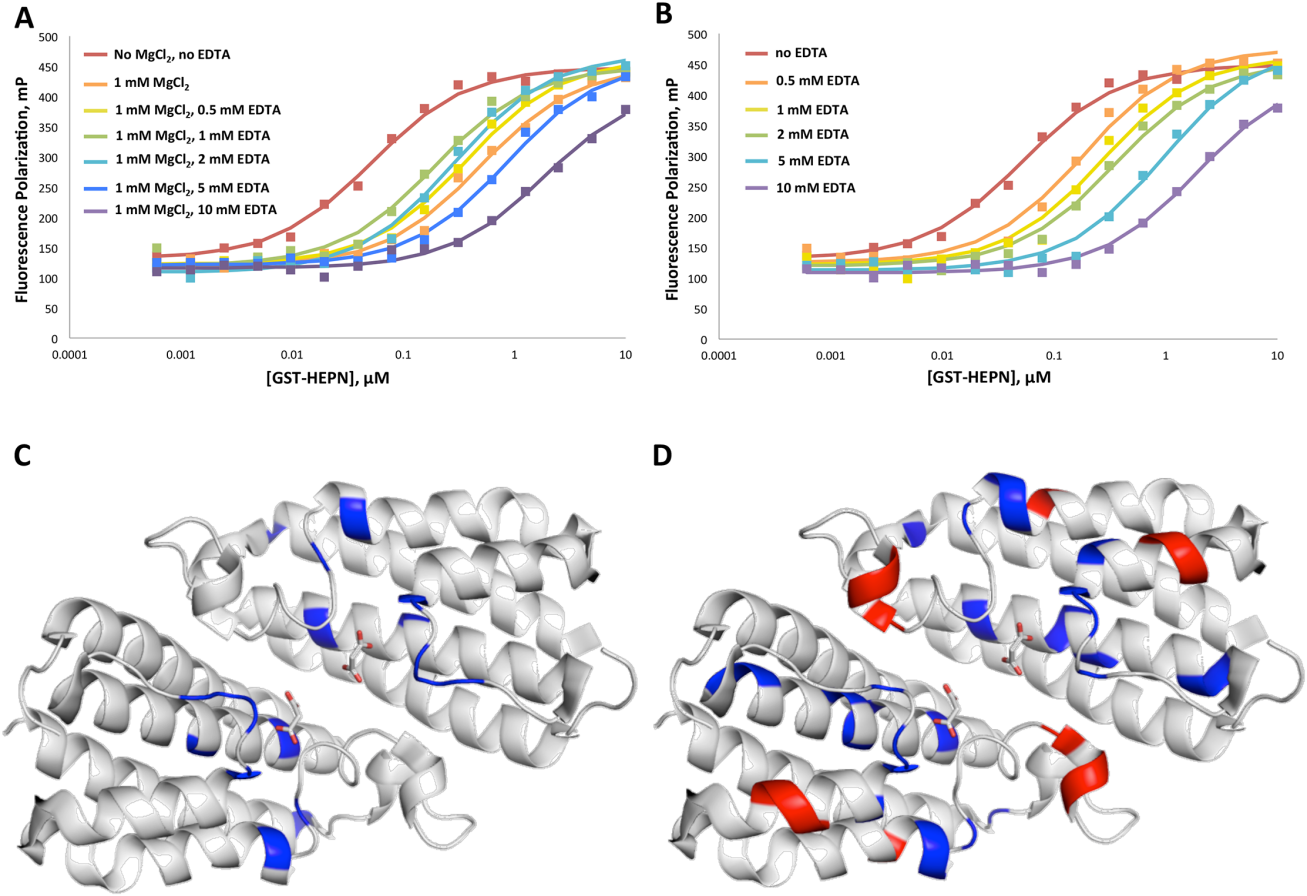
Effects of MgCl_2_ and EDTA on the interaction between F12-ATP and GST-HEPN. A. F12-ATP vs. GST-HEPN FP titration was carried out in the presence of 1 mM MgCl_2_ and increasing concentrations of EDTA. B. F12-ATP vs. GST-HEPN FP titration with increasing concentrations of EDTA alone. C. EDTA-interacting residues are labeled in *blue* on the HEPN crystal structure. D. G-tetra-P-binding residues are mapped onto HEPN. G-tetra-P-binding residues that are also involved in GTP-binding are labeled in *blue* and additional residues only involved in G-tetra-P-binding are labeled in *red*.

Since most nucleotides in the cell form complexes with magnesium ions (Mg^2+^), FP titration between F12-ATP and GST-HEPN was performed in the presence of 1 mM MgCl_2_ (Fig 3A). Comparing to the titration without MgCl_2_, the binding curve right-shifted upon the addition of 1 mM MgCl_2_, showing a 10-fold decrease in affinity. This finding was surprising since most known nucleotide-binding domains require Mg^2+^ for ATP- or GTP-binding. To verify that Mg^2+^ alone was responsible for the decrease in the binding affinity, the experiment was repeated with NaCl. Addition of Cl^-^ had no effect on the binding affinity (data not shown). To confirm the Mg^2+^ effect, the titration was performed in the presence of increasing concentrations of EDTA, a metal chelator, in addition to 1 mM MgCl_2_. While EDTA was able to partially reverse the effect of Mg^2+^ at low concentrations, additions above 1 mM actually decreased the affinity. To investigate the effect of EDTA alone on binding, the titration was repeated in the absence of MgCl_2_. Increasing concentrations of EDTA decreased the binding affinity (Fig 3B). An FP competition assay with fixed concentrations of F12-ATP and GST-HEPN confirmed that EDTA disrupts the binding (S4 Fig). The affinity of EDTA was estimated from the competition curve to be ~510 μM. An NMR titration was performed with ^15^N-HEPN to identify the EDTA binding site (S4B Fig). Mapping the shifted residues onto the HEPN structure confirmed that EDTA interacts with GTP- and malonate ion-binding site (S5 Fig and Fig 3C). Consistent with its ~500-fold weaker affinity than GTP, titration with EDTA resulted in fewer residues showing chemical shift changes than that with GTP. Taken together, the FP and NMR titration assays showed that Mg^2+^ and EDTA both interferes with the nucleotide-binding activity of HEPN, Mg^2+^ most likely by interacting with F12-ATP and EDTA by blocking the GTP-binding pocket on HEPN.

### Nucleotide and nucleotide analog screen

**Fig 4:**
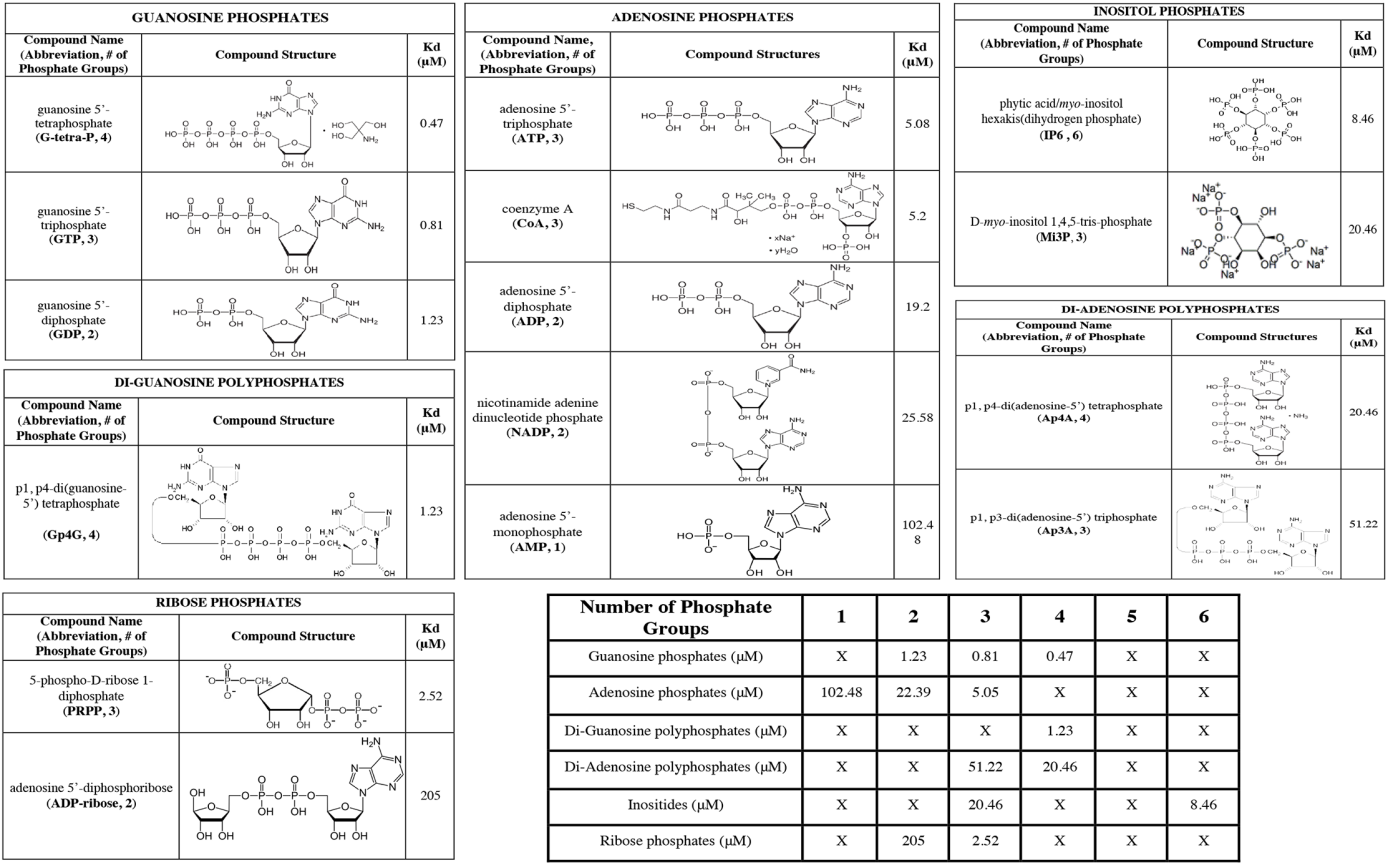
Affinities of nucleotides and nucleotide analogs for HEPN estimated from FP competition assays. The affinities of the compounds were summarized along with their chemical structures.

To further investigate the ligand-binding properties of HEPN, FP competition assays were performed with a variety of nucleotide and nucleotide analogs. The affinities of the compounds for GST-HEPN were determined from the competition curve and summarized in Fig 4. The compounds were grouped based on their chemical moieties and ranked according to the number of phosphate groups they possess. The relative affinities of GTP, GDP, ATP, ADP and AMP correlate well those previously obtained from ITC titrations, validating the assay design. Within each group of the tested compounds, the affinity of a compound for HEPN positively correlates with the number of phosphate groups. Among the compounds with the same number of phosphate groups, guanine nucleotides have higher affinities than adenine nucleotides, which in turn have higher affinities than inositol phosphates and ribose phosphates. For the same number of phosphates, mononucleotides such as guanosine tetraphosphate (G-tetra-P) have higher affinities than dinucleotides such as diguanosine tetraphosphate (Gp4G). Together, the results suggest that HEPN preferentially binds to compounds with a higher number of exposed phosphate groups.

### HEPN interacts with nucleotides via their phosphate groups

Among the compounds tested, G-tetra-P exhibited about two-fold higher affinity for GST-HEPN than GTP. An NMR titration was performed with ^15^N-HEPN and G-tetra-P to investigate how the extra phosphate group on G-tetra-P contributes to its high affinity for HEPN (S6A Fig **)**. Changes in chemical shifts were observed for most GTP-binding residues, as well as additional residues in the vicinity of the binding site (Fig 3D and S6B Fig). Positions of the residues suggest that the extra phosphate group of G-tetra-P can facilitate additional interactions between the compound and HEPN, consistent with our observation that HEPN binds to nucleotides primarily via their phosphate groups.

### High-throughput screen

The FP assay was subsequently adapted into a high-throughput screen in collaboration with the High-throughput Screen Facility at University of Montreal to search for novel small molecular ligands of HEPN. The screen was carried out in a 384-well miniaturized format with 5 nM F12-ATP and 120 nM GST-HEPN in each well. Over 115, 000 compounds were tested and ranked according to the decrease in FP signal upon the addition of the compound. GST-HEPN buffer and GTP were used as negative and positive controls, respectively. The assay was highly robust, with a high Z-factor of 0.80 (S7A Fig). 560 compounds were selected for confirmation.

The hit candidates were then subjected to a counter screen using a peptide binding domain, MLLE, and a fluorescein-labeled peptide to eliminate false positives. None of the candidates showed interference with the peptide-binding assay (data not shown). We then eliminated the candidates belonging to structural groups that frequently show up as false positives in high-throughput screens [19]. Out of the 560 hits from the primary screen, 30 were selected for further verification.

### Hit candidates confirmation

**Fig 5:**
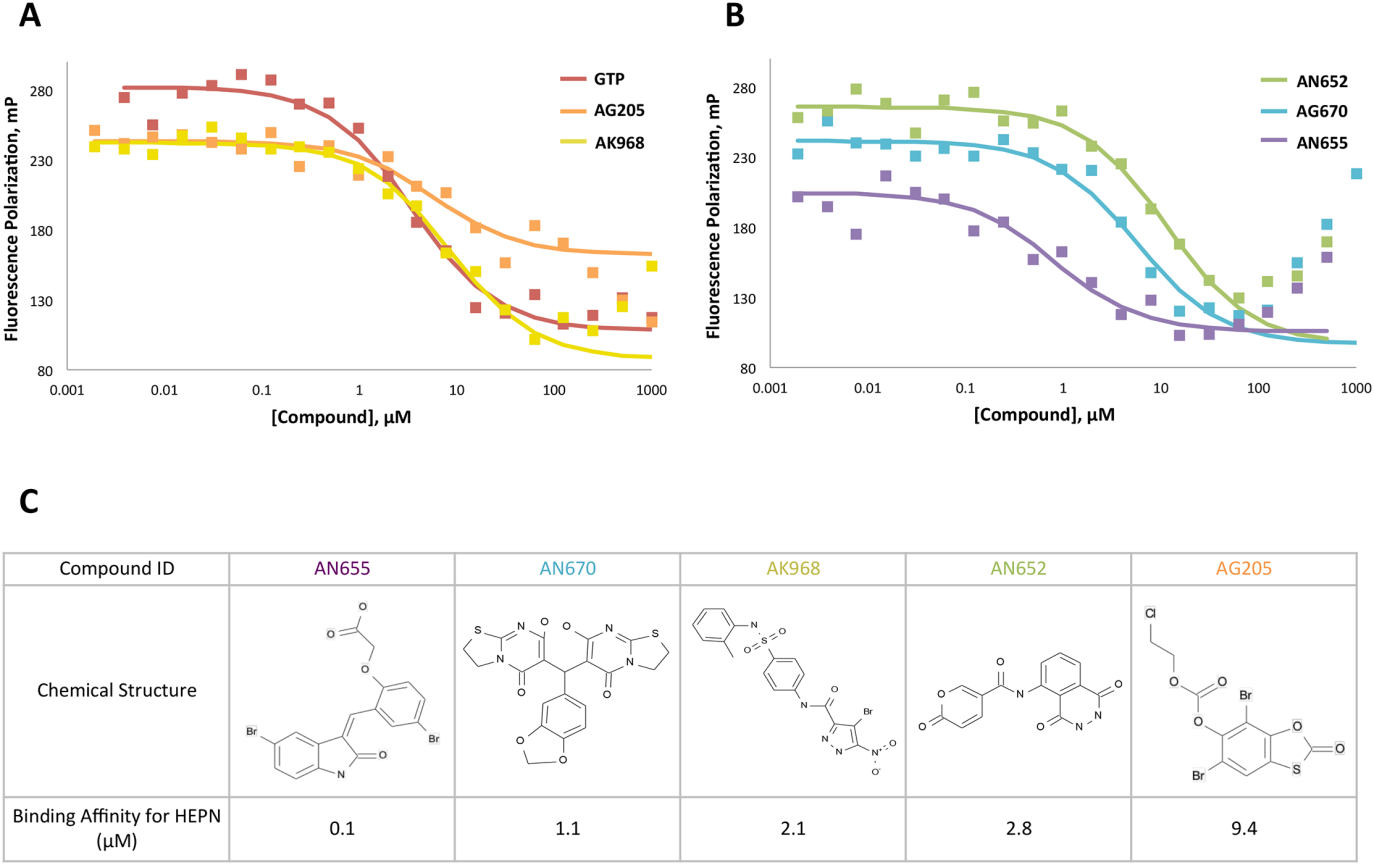
Confirmation of HTS candidates by FP competition assay. A and B. 5 nM F12-ATP and 200 nM GST-HEPN were titrated with the hits to confirm their binding to GST-HEPN. C. Affinities of the compounds for HEPN were estimated from the competition assays and summarized along with their chemical structures.

Five of the thirty hit candidates were purchased from commercial sources for confirmation experiments. The affinities of the hit candidates were then determined to high precision with GTP for comparison (Fig 5). DMSO, which was used as a solvent for the compounds, was shown to have no effect on the FP signal at concentrations below 1% (v/v) (S7B Fig).

**Fig 6:**
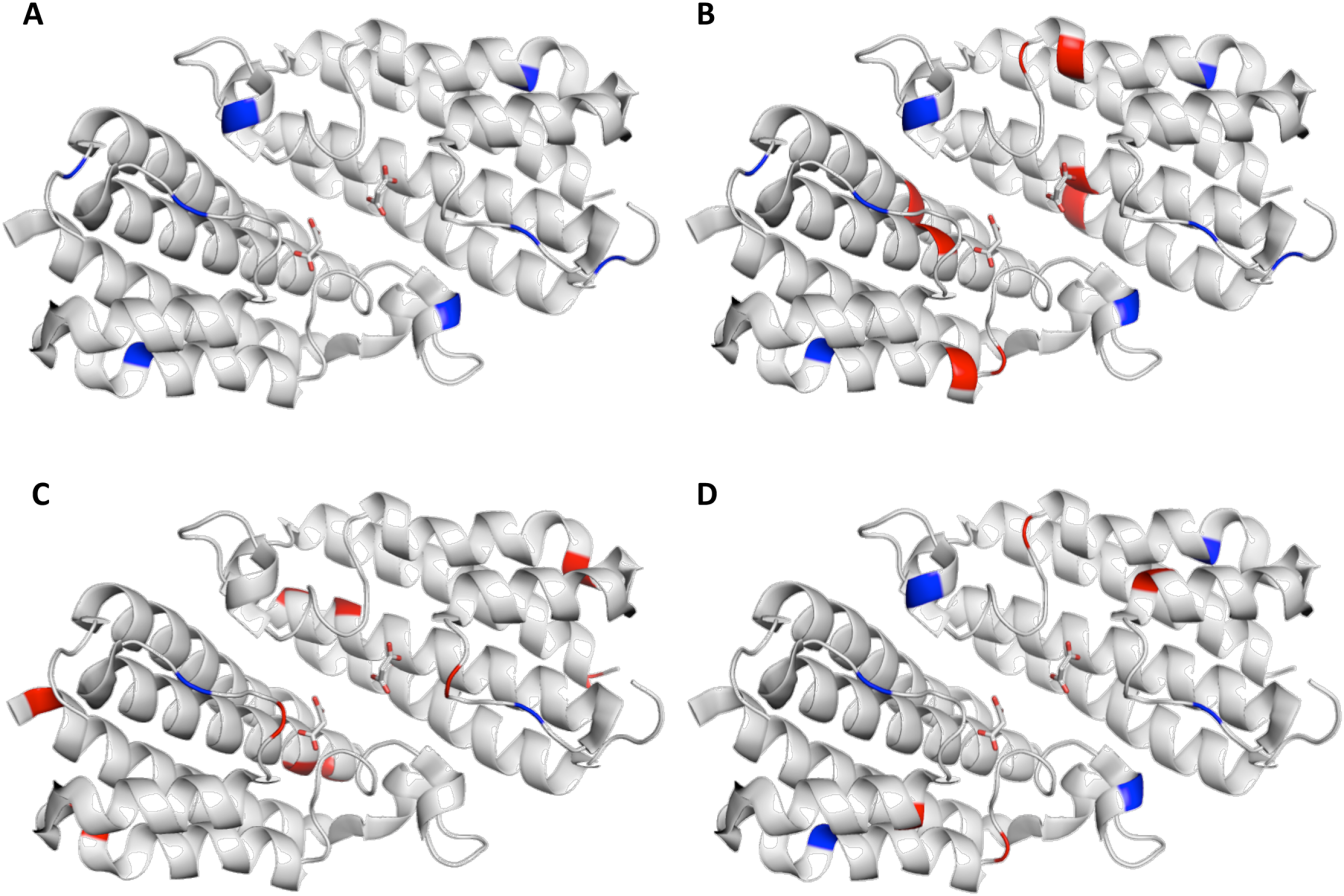
Residues involved in binding mapped onto HEPN crystal structure. Residues that produced chemical shift changes during the NMR titrations with DMSO (A), AN670 (B), AK968 (C) and AN652 (D) were mapped onto the HEPN crystal structure. In each structure, DMSO-binding residues are labeled in *blue* and additional residues binding to the hit candidates are labeled in *red*.

All five compounds showed low micromolar affinities for HEPN. AN-655 exhibited an 8-fold higher affinity for HEPN than GTP. The other four compounds showed either similar or slightly lower affinities than GTP. AN670 exhibited two- and three-fold higher affinity than AK968 and AN652, respectively. Since the decrease in FP signal could also be due to compound autofluorescence or non-specific binding, NMR spectroscopy was used to verify the binding between the compounds and HEPN (S7C and S8 Figs). Binding was observed by NMR for AN670, AN968 and AN652. AN655 did not produce any chemical shift perturbations in the NMR spectrum. Mapping the shifted residues from the DMSO control revealed that DMSO-induced chemical shift changes in a few residues at the exposed surfaces of HEPN (Fig 6A and S9A Fig). AN670 did not interact with the loops at the GTP-binding site, but bound to residues deeper within the pocket (Fig 6B and S9B Fig). AK968 primarily interacted with the loops at the binding site and AN652 seemed to only interact with a few residues at the exposed surface of the pocket (Fig 6C and 6D and S10 Fig).

## Discussion

We developed an FP assay with the HEPN domain of sacsin and fluorescein-labeled ATP to investigate the ligand-binding properties of sacsin. Both Mg^2+^ and EDTA disrupt the interaction between F12-ATP and GST-HEPN, Mg^2+^ most likely by binding to F12-ATP and EDTA by binding to GST-HEPN. The FP competition assay performed with nucleotides and nucleotide analogs revealed that HEPN preferentially binds to nucleotides with higher number of accessible phosphate groups. An FP high-throughput screen subsequently identified thirty potential ligands for HEPN, three of which were confirmed by NMR titrations.

Most nucleotides in the cell form complexes with Mg^2+^, which coordinates the geometry of β and γ phosphate groups. In many nucleotide-binding proteins, the nitrogenous base of the nucleotide interacts with the binding site. The presence of Mg^2+^ is usually either required or advantageous for binding [20]. In the case of HEPN, however, Mg^2+^ disrupts the binding of F12-ATP, which suggests that the binding mechanism differs from conventional nucleotide-binding domains. The nucleotide and nucleotide analog screen revealed a positive correlation between the number of accessible phosphate groups on a compound and its affinity for HEPN. G-tetra-P, which has one more phosphate group than GTP, showed twice the affinity of GTP. NMR titration confirmed that G-tetra-P forms additional interactions with residues near the nucleotide-binding pocket. Together, these observations suggest that HEPN binds to nucleotides via electrostatic interactions with their phosphate groups.

Nevertheless, HEPN exhibits some degree of specificity to the chemical moiety attached to the phosphate groups. For instance, NMR titration of HEPN with F12-ATP suggests that the fluorescein on F12-ATP likely interacts with residues near the nucleotide-binding site, which contributes to its 65-fold higher affinity relative to ATP. GTP also exhibits approximately 6-folder higher affinity than ATP. The preference for a guanine base was conserved among the nucleotides tested: Gp4G bound better than the analogous di-adenosine tetraphosphate, Ap4A.

Although the chemical structures of the five hit candidates from the high-throughput screen suggest that they are hydrophobic, AN670, AK968 and AN652 have a larger number of electron-rich nitrogen (N) and oxygen (O) atoms that could readily form hydrogen bonds with residues on HEPN. In contrast, AN655 has fewer N and O atoms and did not interact with the nucleotide-binding pocket on HEPN as well as the other three compounds. The decrease in FP signal observed in the AN655 competition assay could be due to light scattering from protein or AN655 aggregation.

Although the nucleotide-binding pocket on HEPN is symmetrical, the domain binds GTP and ATP, which are asymmetrical chemical structures, with a stoichiometry of one nucleotide to one HEPN dimer [15]. The crystal structure of HEPN contains two malonate ions, present at high concentration in the crystallization buffer, symmetrically arranged in the nucleotide-binding site. Among the high affinity ligands identified from the high-throughput screen, only AN670 is symmetrical. The NMR titration showed that AN670 is able to interact with a number of residues deep into the nucleotide-binding pocket on HEPN. Nevertheless, AN670 only exhibits two- to three-fold higher affinity than the other two HTS hits AK968 and AN652. The only other symmetrical ligand, EDTA, shows weak affinity. The carboxylic acid groups of EDTA are too close together to bridge the malonate ions observed in the HEPN crystal. Although no compounds with multiple carboxylic acid groups were identified among the HTS hits, it is possible that symmetrical, high-affinity ligands can be designed based on the positions of the malonate ions in the crystal structure.

Results from the HTS suggest that the physiological substrate of HEPN is yet to be identified. Kanamycin nucleotidyl-transferase, which contains a C-terminal HEPN domain, also forms a symmetric dimer. A large pocket is formed by the dimerization of both the N- and C-terminal domains of the protein and binds two ATP and kanamycin molecules. It is possible that other domains in sacsin (DnaJ, SIRPTs or UbL) cooperate with the HEPN domain to form a larger binding pocket with greater substrate-binding specificity. Given the putative role of sacsin as a chaperone, negatively charged amino acids in a client protein are possible ligands. HEPN domains have been suggested to bind RNA molecules [8, 9], which is consistent with the strong bias for compounds with multiple phosphate groups observed in the present study. Nevertheless, the identification of small, drug-like ligands of the sacsin HEPN domain opens the door to testing the function of the HEPN domain in cells and a better understanding the molecular pathology of ARSACS.

## Supporting Information

S1 Fig Detection wavelength optimization for MABA-ATP and F12-ATP. A. Absorbance spectra of 0.2 μM MABA-ATP and 3.3 nM F12-ATP at fixed emission wavelength (436 nm for MABA-ATP and 520 nm for F12-ATP). The optimal excitation wavelengths of MABA-ATP and F12-ATP were 338 nm and 490 nm, respectively. B. Emission spectra of MABA-ATP and F12-ATP fixed excitation wavelength (338 nm for MABA-ATP and 490 nm for F12-ATP). The optimal emission wavelengths of MABA-ATP and F12-ATP were 436 nm and 520 nm, respectively.
(TIF)

S2 Fig GST control titration. 0.5 μM MABA-ATP and 5 nM F12-ATP were titrated against various concentrations of GST to verify whether GST contributes to the observed FP signal in the presence of GST-HEPN. GST did not show binding to either probe at concentrations corresponding to those used in the GST-HEPN titrations.
(TIF)

S3 Fig GTP and F12ATP NMR chemical shift perturbations. A. Weighted sum of ^1^H and ^15^N chemical shift perturbations upon addition of 100 μM GTP to 200 μM ^15^N-HEPN plotted against residue number. B. Weighted sum of chemical shift perturbations upon addition of 110 μM F12ATP to 110 μM ^15^N-HEPN plotted against residue number.
(TIF)

S4 Fig Characterizing the binding between EDTA and GST-HEPN. A. 5 nM F12-ATP and 120 nM GST-HEPN were titrated against EDTA. The affinity of EDTA for GST-HEPN was estimated to be 510 μM. B. Overlay of the ^15^N,^1^H-HSQC NMR spectra of 0.22 mM ^15^N-HEPN alone (*red*) and following the addition of EDTA (0.88 mM in *green* and 1.76 mM in *blue*).
(TIF)

S5 Fig EDTA NMR chemical perturbations. Chemical shift perturbations upon addition of 1.76 mM EDTA to 0.22mM ^15^N-HEPN plotted against residue number.
(TIF)

S6 Fig Identification of G-tetra-P-binding residues on HEPN. **A.** Overlay of the ^15^N,^1^H-HSQC NMR spectra of 390 μM ^15^N-HEPN alone (*red*) and following the addition of G-tetra-P (97.5 μM in *green* and 195 μM in *blue*). B. Chemical shift perturbations upon addition of 195 μM G-tetra-P to 390 μM ^15^N-HEPN plotted against residue number.
(TIF)

S7 Fig High-throughput screen. A. Z-Factor distribution of the primary screen. Z-factor from each compound plate was plotted against the plate number. The average Z-factor for the screen was ~0.80, indicating that the assay was highly robust. B. DMSO control for FP competition assay. 5 nM F12-ATP and 200 nM GST-HEPN were titrated against various percentages of DMSO as negative control. The highest %DMSO in FP competition assays with the hit candidates was around 1%. C. Overlay of the ^15^N,^1^H-HSQC NMR spectra of 100 μM ^15^N-HEPN alone (*red*) and following the addition of DMSO (3% *green* and 7% *blue*). The highest %DMSO in NMR titrations with hit candidates was 7%.
(TIF)

S8 Fig Hit candidate confirmation by NMR titration. A. Overlay of the ^15^N,^1^H-HSQC NMR spectra of 100 μM ^15^N-HEPN alone (*red*) and following the addition of DMSO (3% *green* and 7% *blue*). B. Overlay of the spectra of 100 μM ^15^N-HEPN alone (*red*) and following the addition of AN670 (200 μM *green* and 400 μM *blue*). C. Overlay of the spectra of 100 μM ^15^N-HEPN alone (*red*) and following the addition of AK968 (50 μM *green* and 100 μM *blue*). D. Overlay of the spectra of 100 μM ^15^N-HEPN alone (*red*) and following the addition of AN652 (100 μM *green* and 200 μM *blue*).
(TIF)

S9 Fig DMSO and AN670 NMR chemical shift perturbations. A. Chemical shift perturbations upon addition of DMSO (7% by volume) to 100 μM ^15^N-HEPN plotted against residue number. B. Chemical shift perturbations upon addition of 400 μM AN670 to 100 μM ^15^N-HEPN plotted against residue number.
(TIF)

S10 Fig AK968 and AN652 NMR chemical shift perturbations. A. Chemical shift perturbations upon addition of 100 μM AK968 to 100 μM ^15^N-HEPN plotted against residue number. B. Chemical shift perturbations upon addition of 200 μM AN652 to 100 μM ^15^N-HEPN plotted against residue number.
(TIF)
