# Supplementary figures and images for: High-Throughput Screening for Ligands of the HEPN Domain of Sacsin
Source: PLoS One. 2015 Sep 14;10(9):e0137298. doi: 10.1371/journal.pone.0137298 (PMC4569058; doi:10.1371/journal.pone.0137298)

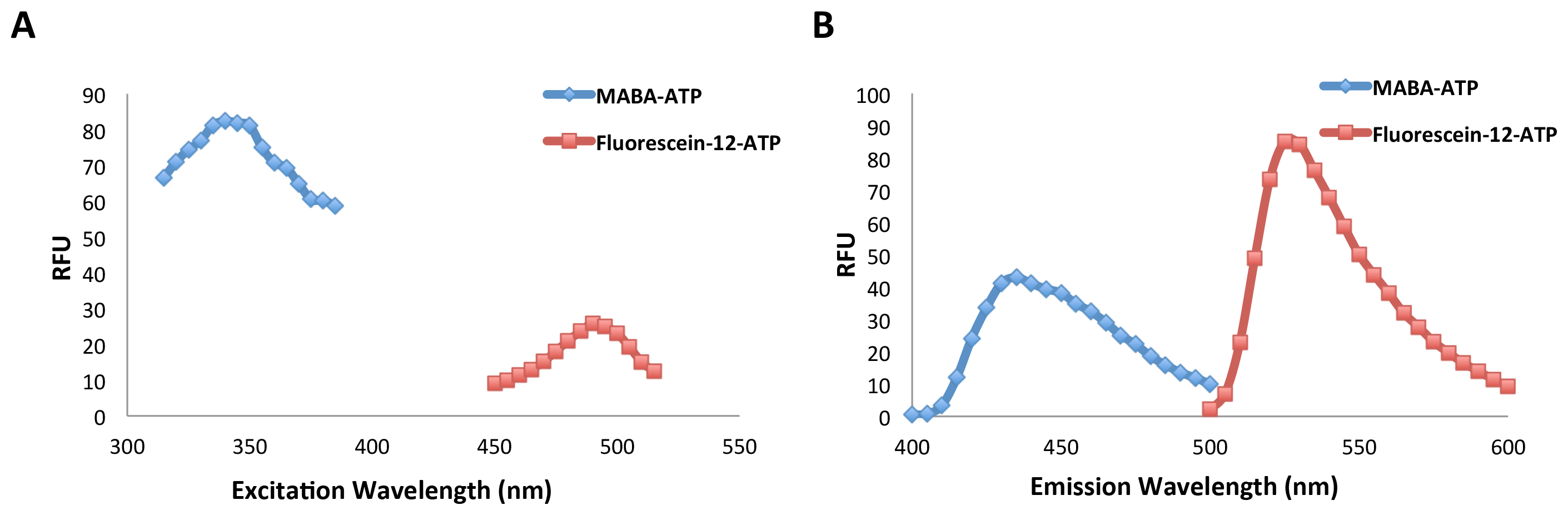

Supplement: S1 Fig — A. Absorbance spectra of 0.2 μM MABA-ATP and 3.3 nM F12-ATP at fixed emission wavelength (436 nm for MABA-ATP and 520 nm for F12-ATP). The optimal excitation wavelengths of MABA-ATP and F12-ATP were 338 nm and 490 nm, respectively. B. Emission spectra of MABA-ATP and F12-ATP fixed excitation wavelength (338 nm for MABA-ATP and 490 nm for F12-ATP). The optimal emission wavelengths of MABA-ATP and F12-ATP were 436 nm and 520 nm, respectively. (TIF) [file pone.0137298.s001.tif]

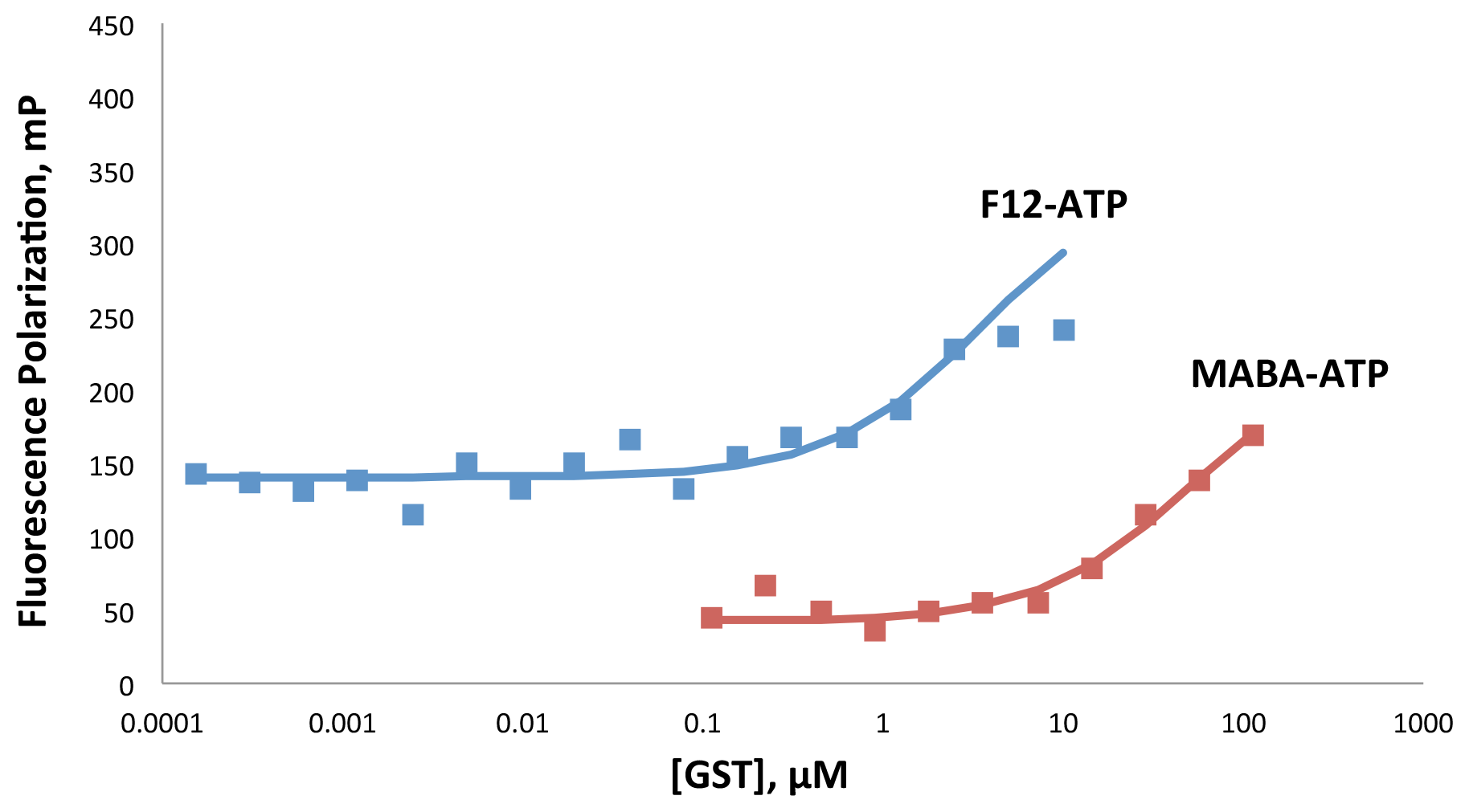

Supplement: S2 Fig — 0.5 μM MABA-ATP and 5 nM F12-ATP were titrated against various concentrations of GST to verify whether GST contributes to the observed FP signal in the presence of GST-HEPN. GST did not show binding to either probe at concentrations corresponding to those used in the GST-HEPN titrations. (TIF) [file pone.0137298.s002.tif]

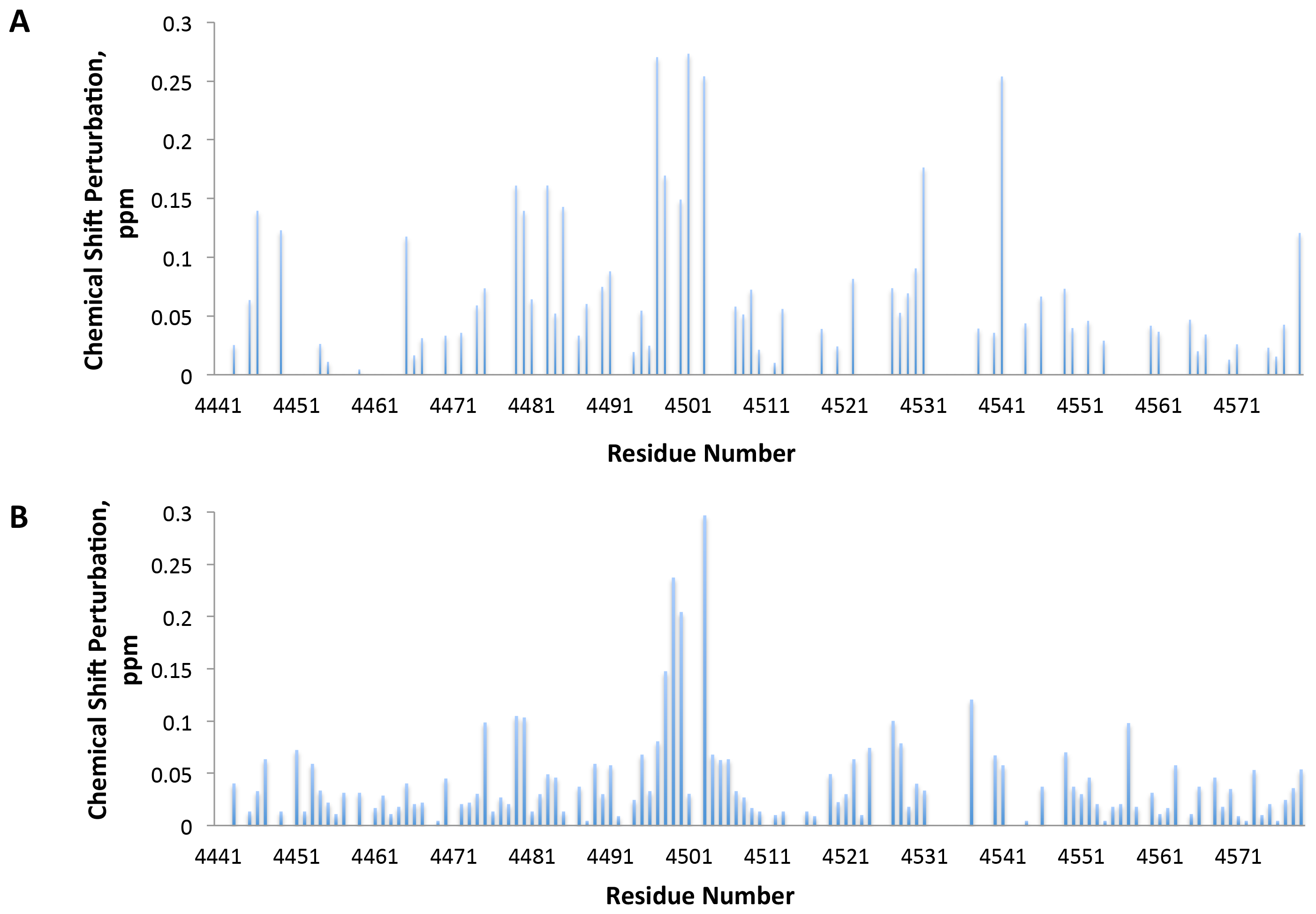

Supplement: S3 Fig — A. Weighted sum of 1H and 15N chemical shift perturbations upon addition of 100 μM GTP to 200 μM 15N-HEPN plotted against residue number. B. Weighted sum of chemical shift perturbations upon addition of 110 μM F12ATP to 110 μM 15N-HEPN plotted against residue number. (TIF) [file pone.0137298.s003.tif]

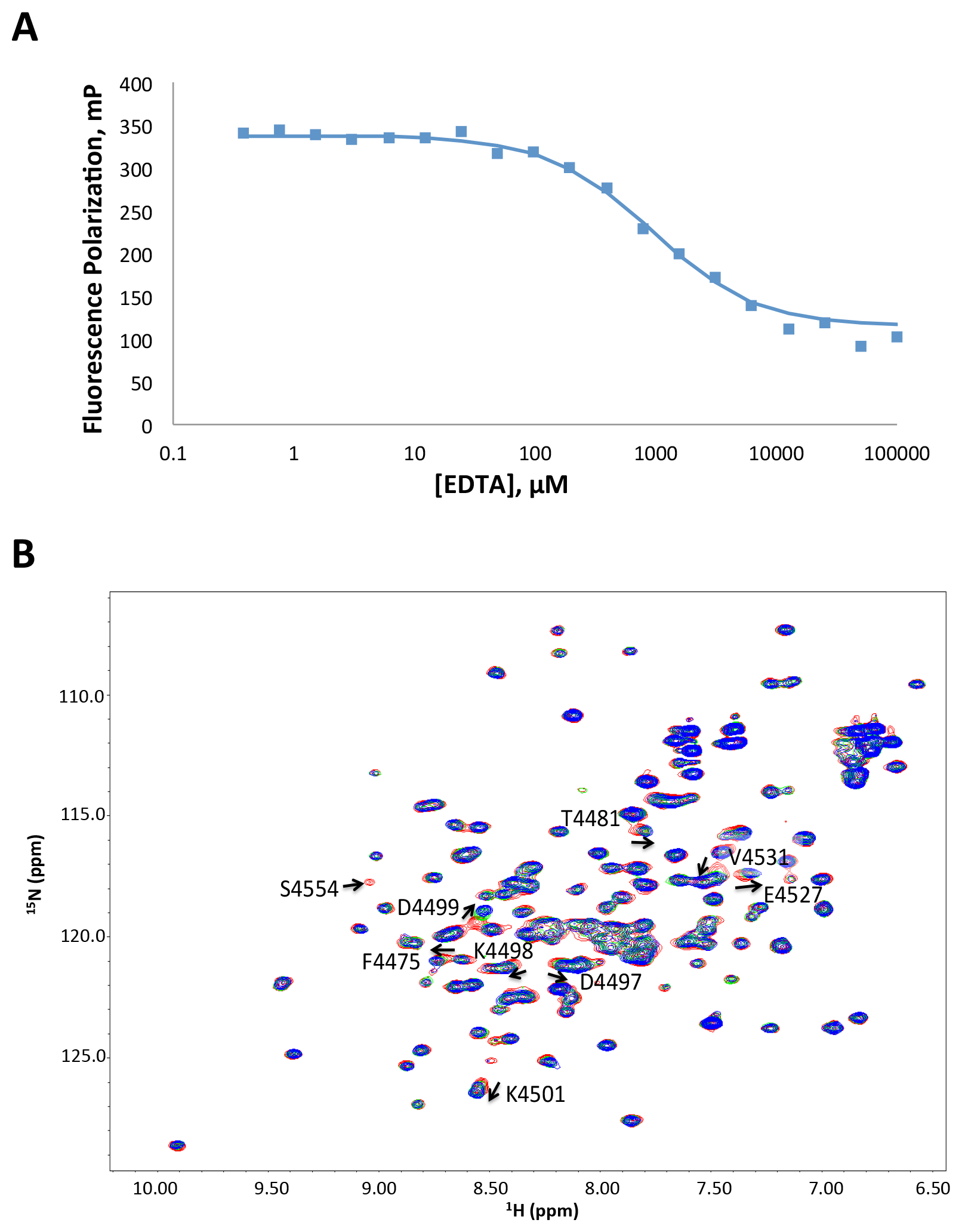

Supplement: S4 Fig — A. 5 nM F12-ATP and 120 nM GST-HEPN were titrated against EDTA. The affinity of EDTA for GST-HEPN was estimated to be 510 μM. B. Overlay of the 15N,1H-HSQC NMR spectra of 0.22 mM 15N-HEPN alone (red) and following the addition of EDTA (0.88 mM in green and 1.76 mM in blue). (TIF) [file pone.0137298.s004.tif]

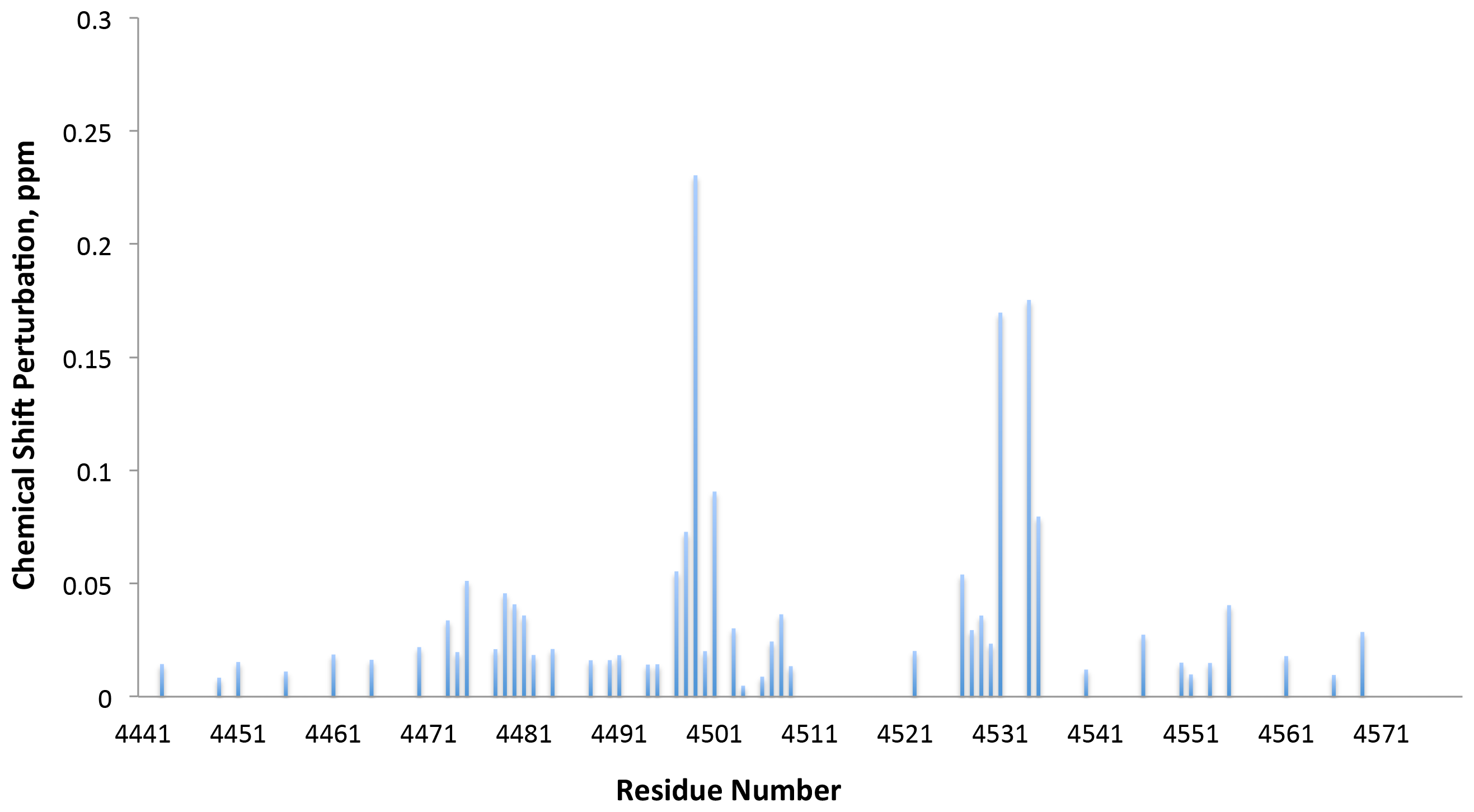

Supplement: S5 Fig — Chemical shift perturbations upon addition of 1.76 mM EDTA to 0.22mM 15N-HEPN plotted against residue number. (TIF) [file pone.0137298.s005.tif]

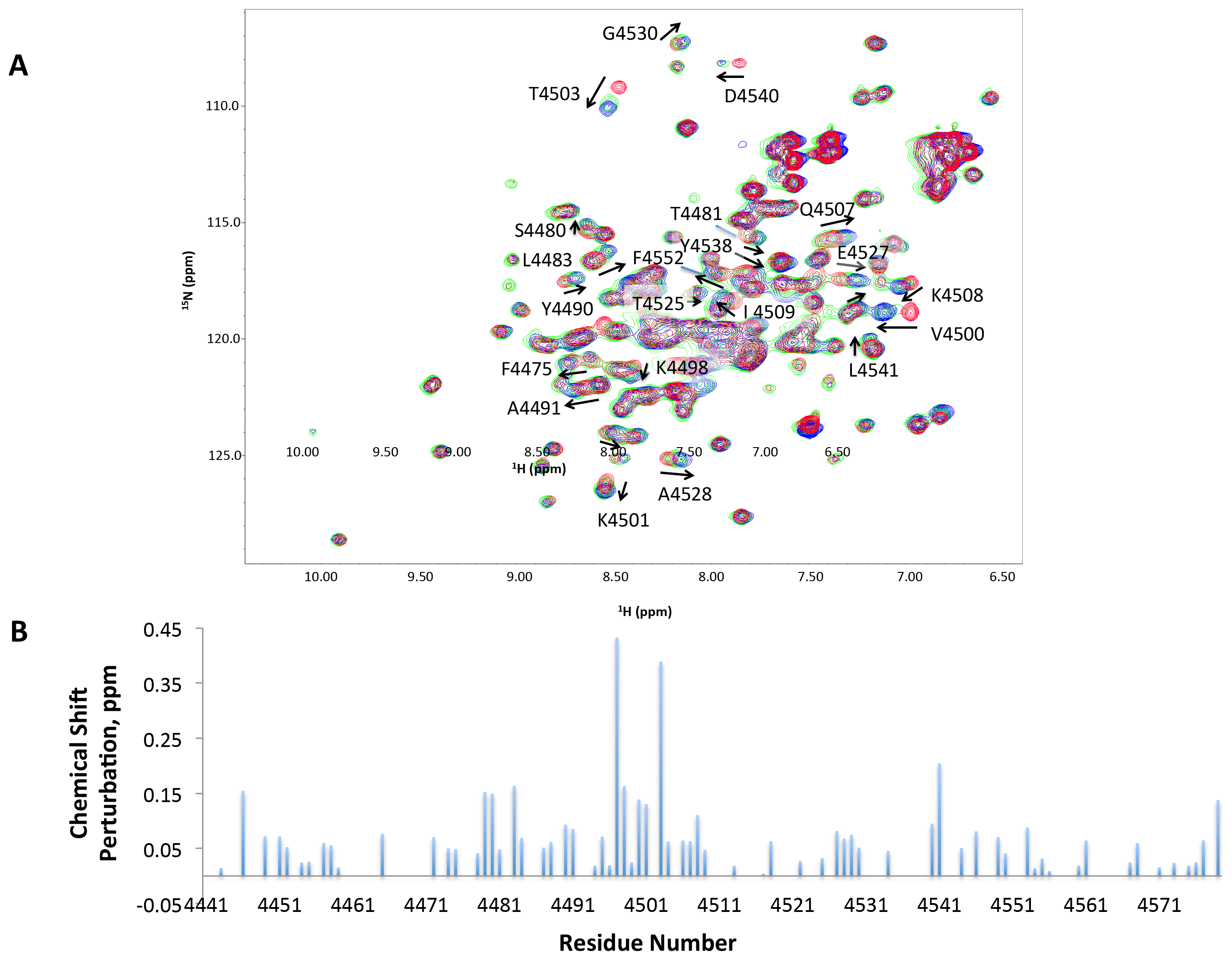

Supplement: S6 Fig — A. Overlay of the 15N,1H-HSQC NMR spectra of 390 μM 15N-HEPN alone (red) and following the addition of G-tetra-P (97.5 μM in green and 195 μM in blue). B. Chemical shift perturbations upon addition of 195 μM G-tetra-P to 390 μM 15N-HEPN plotted against residue number. (TIF) [file pone.0137298.s006.tif]

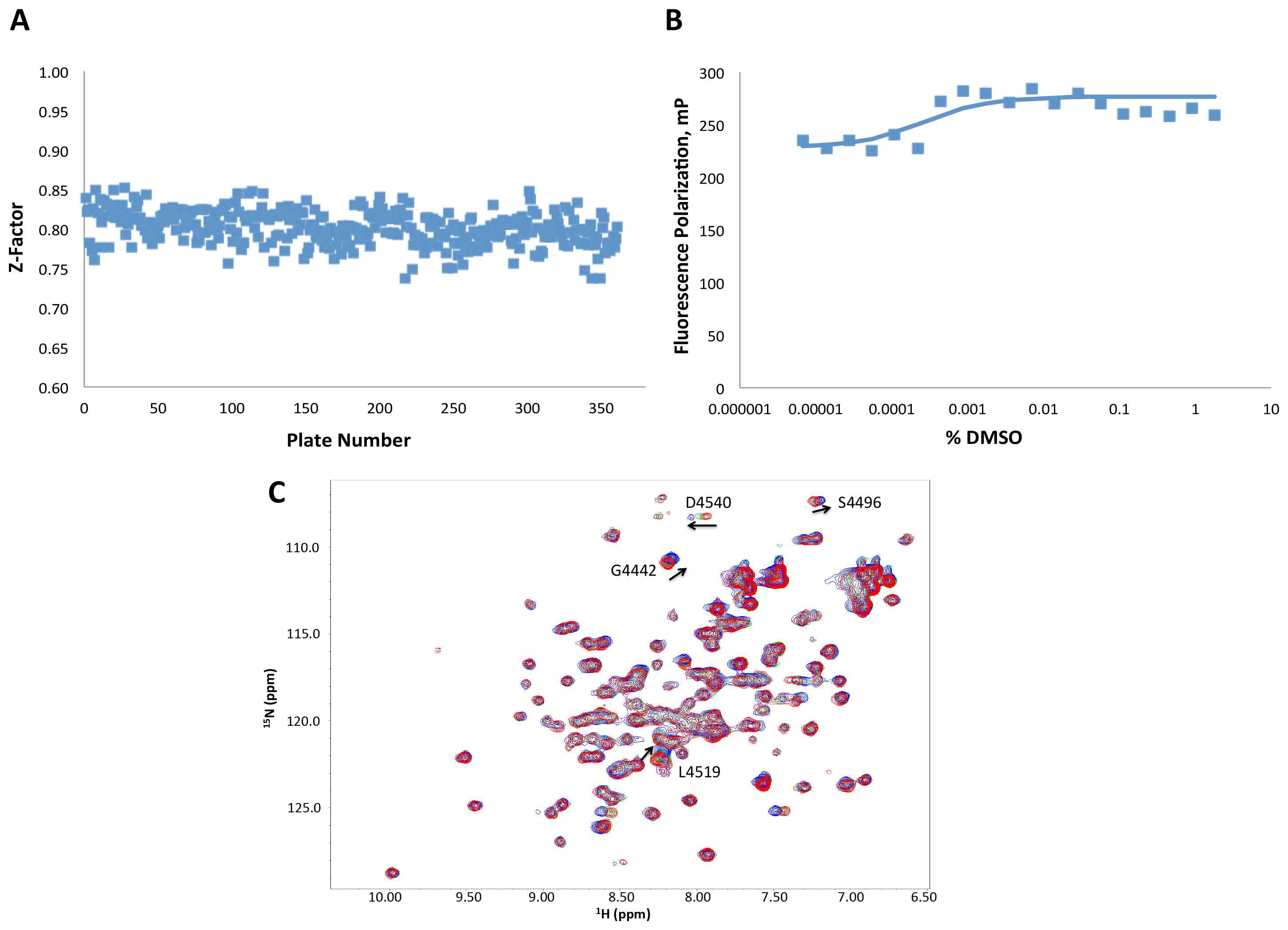

Supplement: S7 Fig — A. Z-Factor distribution of the primary screen. Z-factor from each compound plate was plotted against the plate number. The average Z-factor for the screen was ~0.80, indicating that the assay was highly robust. B. DMSO control for FP competition assay. 5 nM F12-ATP and 200 nM GST-HEPN were titrated against various percentages of DMSO as negative control. The highest %DMSO in FP competition assays with the hit candidates was around 1%. C. Overlay of the 15N,1H-HSQC NMR spectra of 100 μM 15N-HEPN alone (red) and following the addition of DMSO (3% green and 7% blue). The highest %DMSO in NMR titrations with hit candidates was 7%. (TIF) [file pone.0137298.s007.tif]

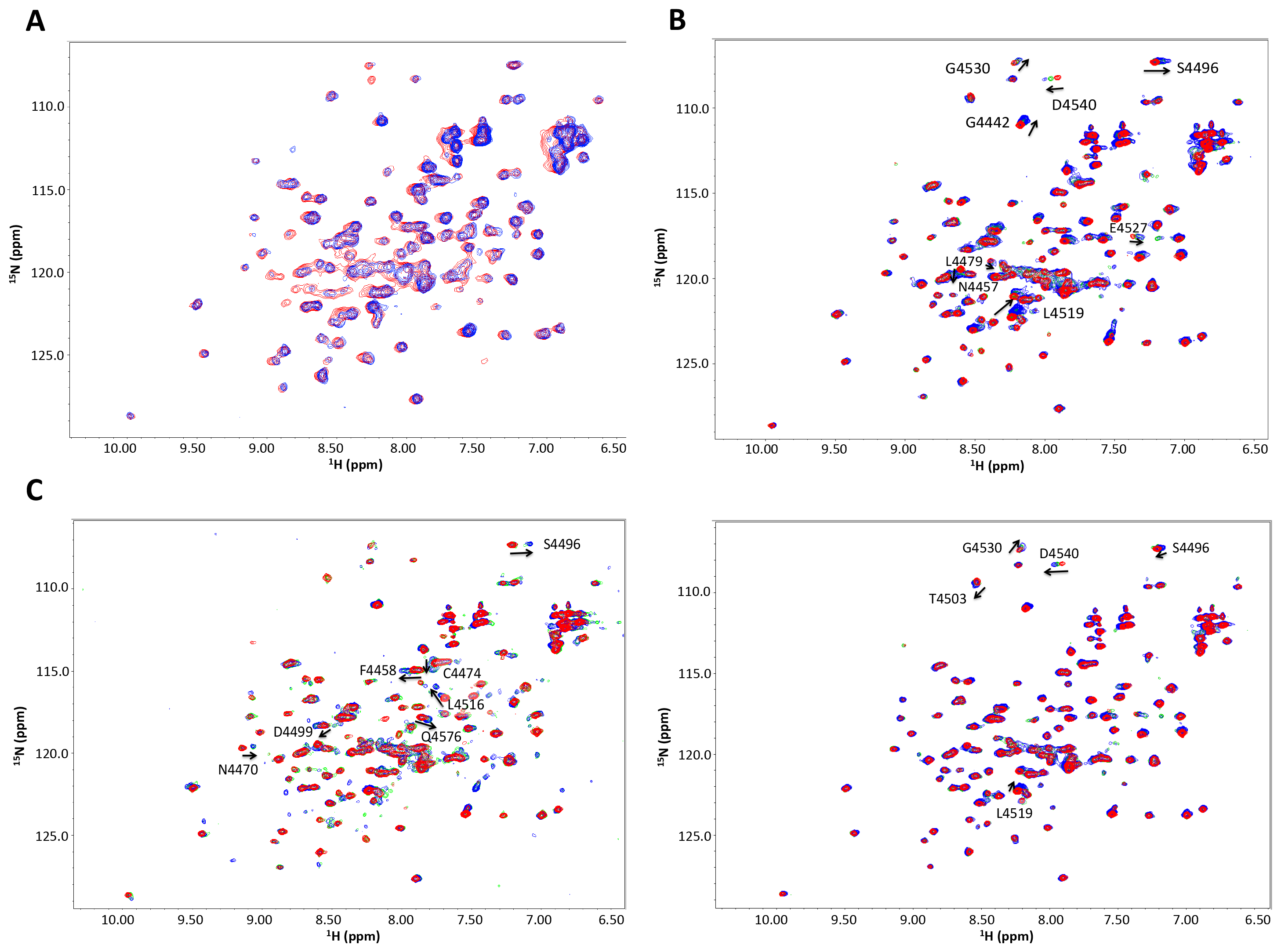

Supplement: S8 Fig — A. Overlay of the 15N,1H-HSQC NMR spectra of 100 μM 15N-HEPN alone (red) and following the addition of DMSO (3% green and 7% blue). B. Overlay of the spectra of 100 μM 15N-HEPN alone (red) and following the addition of AN670 (200 μM green and 400 μM blue). C. Overlay of the spectra of 100 μM 15N-HEPN alone (red) and following the addition of AK968 (50 μM green and 100 μM blue). D. Overlay of the spectra of 100 μM 15N-HEPN alone (red) and following the addition of AN652 (100 μM green and 200 μM blue). (TIF) [file pone.0137298.s008.tif]

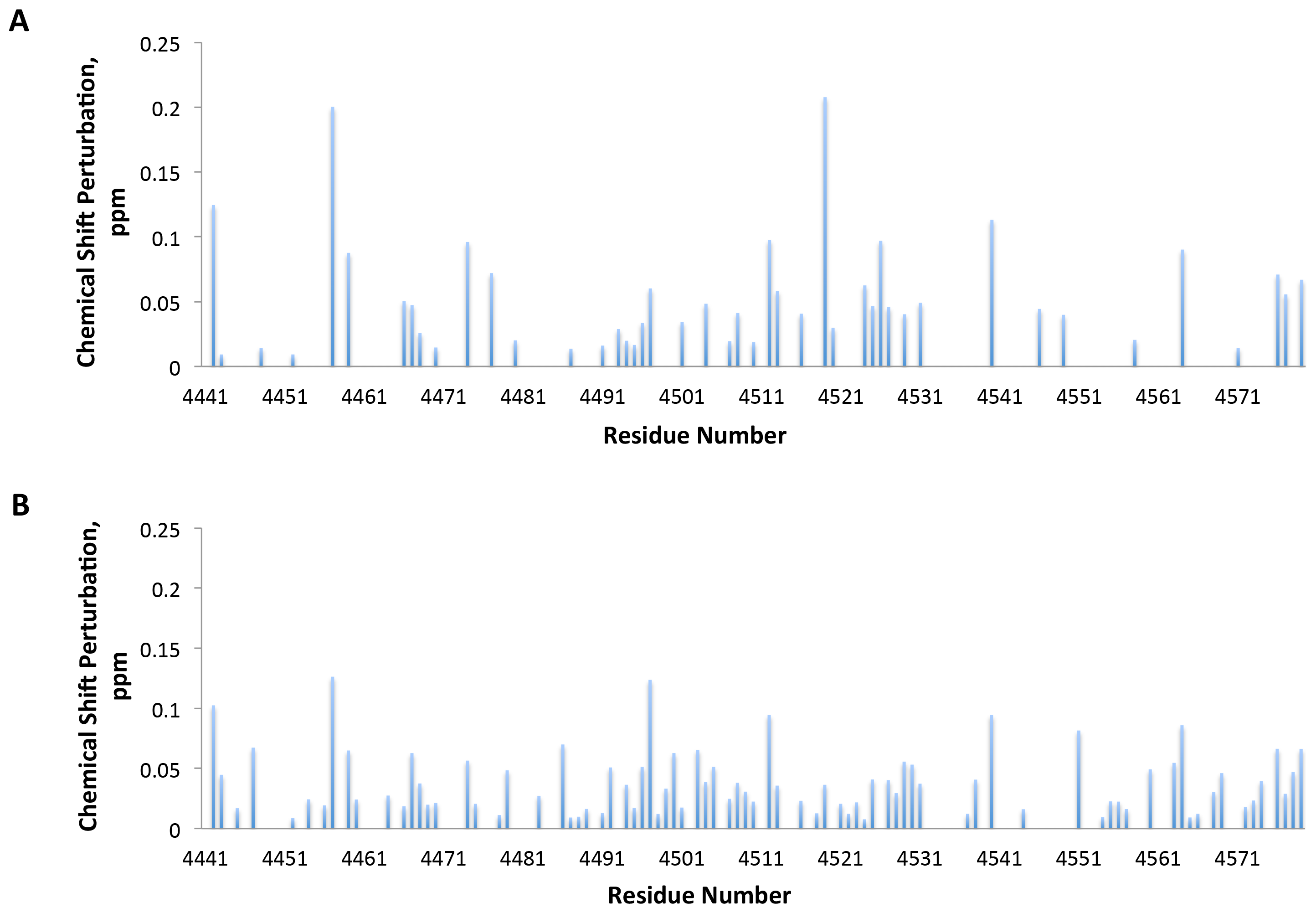

Supplement: S9 Fig — A. Chemical shift perturbations upon addition of DMSO (7% by volume) to 100 μM 15N-HEPN plotted against residue number. B. Chemical shift perturbations upon addition of 400 μM AN670 to 100 μM 15N-HEPN plotted against residue number. (TIF) [file pone.0137298.s009.tif]

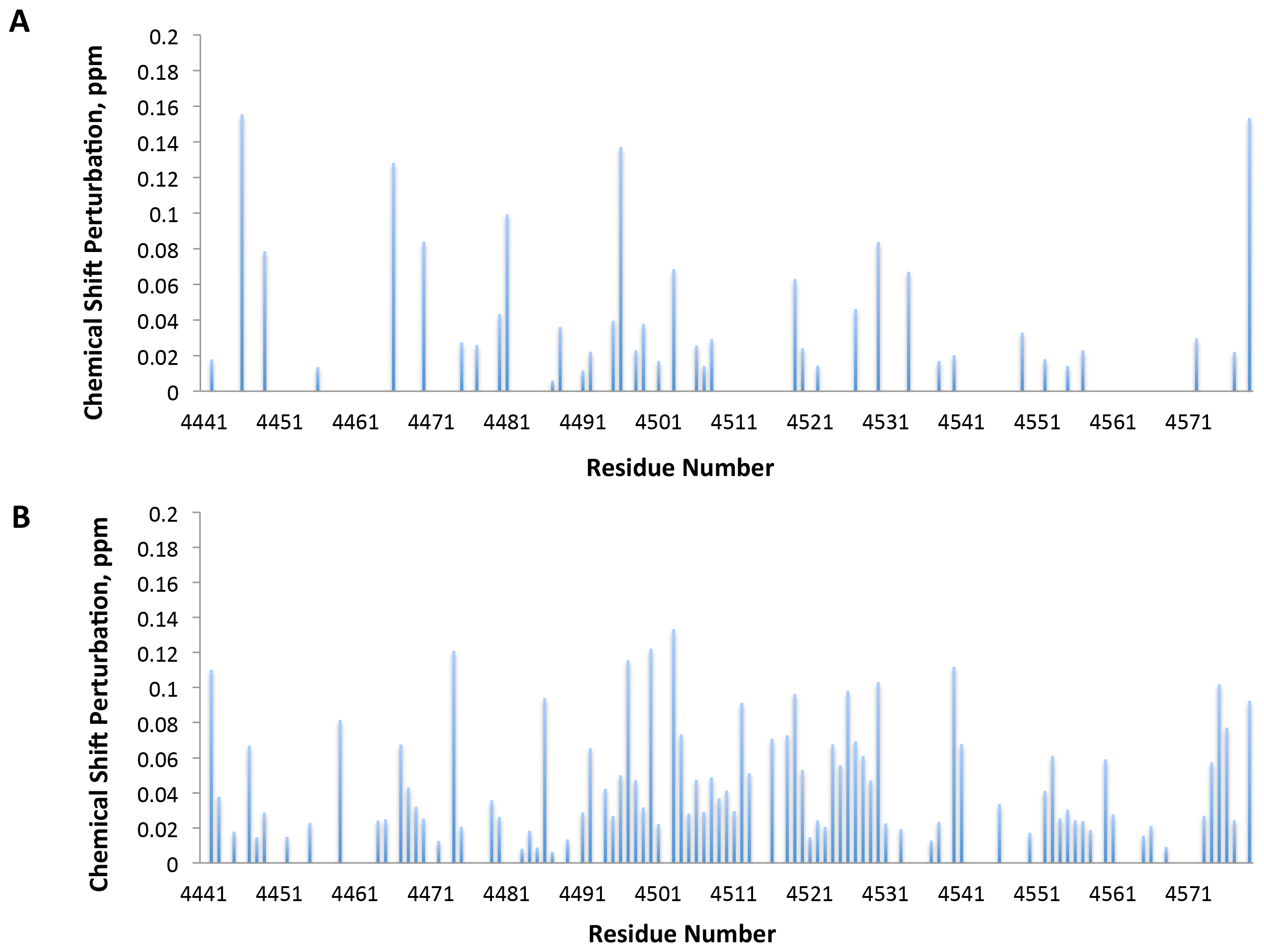

Supplement: S10 Fig — A. Chemical shift perturbations upon addition of 100 μM AK968 to 100 μM 15N-HEPN plotted against residue number. B. Chemical shift perturbations upon addition of 200 μM AN652 to 100 μM 15N-HEPN plotted against residue number. (TIF) [file pone.0137298.s010.tif]
